# Separation of Anti-Proliferation and Anti-Apoptotic Functions of Retinoblastoma Protein through Targeted Mutations of Its A/B Domain

**DOI:** 10.1371/journal.pone.0000082

**Published:** 2006-12-20

**Authors:** B. Nelson Chau, Chris W. Pan, Jean Y.J. Wang

**Affiliations:** Department of Medicine, Division of HematologyOncology, MooresUCSD Cancer Center, University of California San Diego La Jolla, California, United States of America; Sanofi-Aventis, United States of America

## Abstract

**Background:**

The human retinoblastoma susceptibility gene encodes a nuclear phosphoprotein RB, which is a negative regulator of cell proliferation. The growth suppression function of RB requires an evolutionarily conserved A/B domain that contains two distinct peptide-binding pockets. At the A/B interface is a binding site for the C-terminal trans-activation domain of E2F. Within the B-domain is a binding site for proteins containing the LxCxE peptide motif.

**Methodology/Principle Findings:**

Based on the crystal structure of the A/B domain, we have constructed an RB-K530A/N757F (KN) mutant to disrupt the E2F- and LxCxE-binding pockets. The RB-K530A (K) mutant is sufficient to inactivate the E2F-binding pocket, whereas the RB-N757F (N) mutant is sufficient to inactivate the LxCxE-binding pocket. Each single mutant inhibits cell proliferation, but the RB-KN double mutant is defective in growth suppression. Nevertheless, the RB-KN mutant is capable of reducing etoposide-induced apoptosis.

**Conclusion/Significance:**

Previous studies have established that RB-dependent G1-arrest can confer resistance to DNA damage-induced apoptosis. Results from this study demonstrate that RB can also inhibit apoptosis independent of growth suppression.

## Introduction

The retinoblastoma susceptibility gene (*RB1*) encodes a nuclear phosphoprotein RB with tumor suppression function [1]. Inheritance of germline *RB1* mutation causes retinoblastoma with 90% penetrance in children; the tumor cells exhibit loss of heterozygosity (LOH) at the *RB1* locus with the invariable loss the normal *RB1* allele [2], [3]. The bi-alleleic inactivation of the *RB1* gene has also been detected in sporadic human cancers of a variety of tissue origins at an average frequency of approximately 10% (COSMIC database at the Sanger Genome Center). The current knowledge suggests that RB suppresses tumor development by inhibiting cell proliferation and promoting terminal differentiation [1]. The anti-proliferation function of RB is dependent on its interaction with the cellular E2F-family transcription factors, which are heterodimers consisting of E2F and DP subunits [4]. RB directly interacts with several members of the E2F family to inhibit E2F-dependent transcription [4]. The E2F transcription factors regulate genes required for cell proliferation and apoptosis [5]. By inhibiting E2F-dependent transcription, RB negatively regulates cell proliferation and apoptosis.

The growth suppression function of RB protein requires its A/B domain that is conserved in the RB-family proteins. The A/B domain of the human RB protein contains at least two distinct peptide-binding pockets, whose structures have been elucidated by X-ray crystallography [6], [7]. The E2F-peptide binding pocket resides at the A/B domain interface, which binds the C-terminal peptide of E2F-1, 2, and 3[7]. The LxCxE-peptide binding pocket is a shallow groove within the B-domain, which mediates the interaction with proteins containing the LxCxE peptide motif [6]. The two distinct peptide-binding pockets in the A/B domain have each been inactivated by targeted substitution mutations [8]–[10].

Disruption of the LxCxE-binding pocket abrogates the interaction between RB and viral oncoproteins such as the SV40 T-antigen, the HPV E7 protein and the adenovirus E1A protein [8], [10]. The LxCxE-binding-defective RB mutants retain growth suppression function because these mutants retain their interactions with E2F [8], [10]. One of the LxCxE binding-defective mutants constructed by our lab contains a single substitution mutation of Asn757 (RB-N, N757F), which is sufficient to disrupt the LxCxE-binding pocket [8]. This RB-N mutant represses E2F-dependent transcription, inhibits DNA synthesis, and reduces colony formation [8]. As reported here, we have since disrupted the E2F peptide-binding pocket at the RB A/B interface by mutating Lys530 with Ala (RB-K, K530A). The RB-K mutant also remains competent in inhibiting cell proliferation. However, the RB-KN double mutant does not induce growth arrest.

Previous studies have demonstrated that RB-dependent growth arrest is protective against apoptosis. Fibroblasts derived from *Rb*-null embryos are defective in DNA damage-induced G1, S and G2 arrest and exhibit increased apoptotic response to genotoxins [11], [12]. The ectopic expression of RB in SAOS-2 osteosarcoma cells induces G1 arrest and protects from apoptosis induced by ionizing radiation [13]. Induced expression of a constitutively active RB that lacks nine phosphorylate sites (PSM-RB) interfered with doxorubicin-induced activation of caspase-3 as a consequence of G1-arrest [14], [15]. The apoptosis resistance of G1-arrested cells is likely to involve RB-dependent transcriptional repression of E2F-regulated pro-apoptotic genes [5]. Therefore, the anti-apoptotic activity of RB has mostly been considered as a secondary effect of its growth suppression function. In this study, we show that RB and RB-KN can inhibit apoptosis without causing cell cycle arrest. We have demonstrated that RB-KN is a separation of function mutant, which is useful in further studies of the anti-apoptotic function of RB.

## Materials and Methods

### Cell culture and plasmid construction

Human breast adenocarcinoma MDA-MB468 and osteosarcoma SAOS-2 cells were maintained in DMEM supplemented with 10%FBS. Transfections were performed with Fugene (Roche Diagnostics) as per manufacturer's instructions. K530A and N757F mutations were introduced into human RB cDNA via PCR-mediated mutagenesis and the resulting mutation confirmed by DNA sequencing.

### GST pull-down, co-immunoprecipitation and immunoblotting

GST fusion proteins of RB (human, containing amino acids 395 through 928), E2F1 (human, full-length), E2F3 (human, full-length), DP1 (human, full-length) and E7 (human papilloma virus-16, full-length) were expressed in bacteria and purified on Glutathione Sepharose (Pharmacia) by standard methods [8], [16], [17]. The GST fusion proteins were mixed with cell lysates in binding buffer (25 mM Tris-HCl, pH7.5, 250mM KCl, 0.1% Triton X-100, 0.5 mM EDTA, 0.1 mM DTT) and protease inhibitors (Sigma). After extensive washing in the same binding buffer, proteins retained on the solid phase were eluted by the addition of SDS sample buffer and subjected to SDS-PAGE for fractionation prior to being transferred onto PVDF membrane. RB protein on the blot was detected with an affinity-purified polyclonal antibody raised against human RB [18]. HA-tagged proteins of E2F1/3 were detected with an anti-HA monoclonal antibody (Clone HA.11) obtained from Berkeley Antibody Co. E2F1 (KH95) and cleaved caspase-3 (D175) antibodies were obtained from Santa Cruz and Cell Signaling Technology respectively. Cytochrome *c* antibody was obtained from Pharmingen. For co-immunoprecipitation, cells were lysed in the binding buffer, the lysates clarified by centrifugation, and then incubated with anti-RB or anti-HA. The immune complexes were collected on protein G Sepharose, solubilized with SDS sample buffer for immunoblotting by standard methods.

### Adenovirus infection

Recombinant adenovirus was constructed and amplified using the AdEasy system [19]. Cells were infected at a multiplicity of 100 overnight prior to treatment with etoposide.

### Flat cell formation and BrdU incorporation

SAOS-2 cells were transfected with plasmids encoding RB, RB-K, RB-N or RB-KN and the neomycin resistance gene [16], [18]. The transfected cells were selected with G418 for 14 days, stained with crystal violet, and the giant flat cells counted under a dissection microscope (5× magnification) [16], [20]. Cells were incubated with 10 mM BrdU for 14 hours, fixed, and stained with phycoerythrin-conjugated monoclonal antibody against BrdU (BrdU-PE). The percentage of BrdU-positive fraction was determined by FACS analysis. To determine the non-specific background signal, we carried out FACS analyses using cells that were stained with the PE-conjugated IgG. The BrDU-positive fraction was determined using a gate that excluded the background signal.

### Apoptosis assays

To measure the extent of cytochrome *c* release from mitochondria, cells were harvested and resuspended in Mitobuffer (20 mM HEPES (pH 7.5), 1 mM EGTA, 1 mM EDTA, 10 mM KCl, 1.5 mM MgCl2, 1 mM DTT, 0.1 mM PMSF, 2 µg/ml Leupeptin, 2 µg/ml Pepstatin, 2 µg/ml Aprotinin) and then mechanically ruptured with 25 strokes of pestle in a glass homogenizer placed on ice. The lysates were spun free of nuclei and intact cells at a speed of 500×g. The supernatant was then subjected to centrifugation at 8,000×g to pellet mitochondria. To measure the sub-G1 DNA content, cells were fixed in ice-cold ethanol (70%), re-suspended in phosphate buffered saline containing 40 µg/ml RNase and 20 µg/ml propidium iodide and then subjected to FACS analyses.

## Results

### K530A mutation abrogates RB interaction with the trans-activation domain of E2F

**Figure 1:**
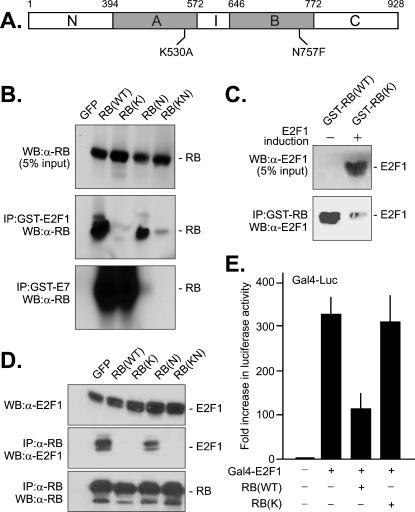
A) Summary of RB mutants. The amino acid numbering is that of the human retinoblastoma protein. Two shaded boxes represent the A and B domains; I, the insert region; N, the region N-terminal to the A domain; C, the region C-terminal to the B domain. B) GST pull-down assay. The RB-deficient MDA-MB468 cells were infected with recombinant adenovirus encoding GFP, RB, RB-K, RB-N or RB-KN, whole cell lysates were incubated with purified GST-E2F1 or GST-E7 fusion protein immobilized on glutathione-Sepharose. The bound fractions were solubilized, fractionated by SDS-PAGE and reacted with an affinity-purified anti-RB. C) Reciprocal GST pull-down assay. Lysates from SAOS-2-tet-E2F1 cells [21] were immunoblotted with anti-E2F1 to demonstrate the induced expression of E2F1 protein (upper panel). Lysates from induced and non-induced cells were incubated with GST-RB or GST-RB-K fusion proteins immobilized on glutathione-Sepharose, and the bound fraction analyzed by immunoblotting with anti-E2F1 (lower panel). D) Co-immunoprecipitation assay. SAOS-2-tet-E2F1 cells [21] were infected with recombinant adenoviruses encoding GFP, RB, RB-K, RB-N or RB-KN and then cultured without tetracycline to induce the expression of E2F1. Co-immunoprecipitation of E2F1 in anti-RB immune complex was determined by immunblotting with anti-E2F1 antibody (α-E2F1). E) Luciferase assay. The Gal4-Luc reporter plasmid contains a firefly luciferase expression cassette with five Gal4 binding sequence motifs. The Gal4-E2F1 protein contains the Gal4-DNA binding domain and the transactivation domain of E2F1 [22]. SAOS-2 cells were transfected with Gal4-Luc and Gal4-E2F1 plasmids in the presence or absence of RB or RB-K. A plasmid expressing Renilla luciferase from the thymidine kinase promoter was included in the transfections for normalization of transfection efficiency. The firefly and Renilla luciferase activities were measured in each sample, the ratio of which was compared among the different transfections with that from the Gal4-Luc only transfected sample as the baseline. The values shown are averages and standard errors from three independent experiments.

The structure of RB A/B domain in complex with the C-terminal E2F peptide (aa 409–426) has been determined [7]. Based on the atomic interactions between RB and E2F amino acids, we constructed several mutations to disrupt this E2F-binding pocket. Among the mutants, Lys530Ala substitution in the A-domain (K530A, RB-K) (Fig. 1A) is sufficient to disrupt the RB-E2F interaction (Fig. 1B–E). We have previously inactivated the LxCxE-binding pocket with the Asn757Phe substitution mutation (N757F, RB-N) in the B-domain (Fig. 1A) [8]. The RB-KN mutant contains both point mutations.

The RB and mutant proteins were expressed in the RB-deficient breast cancer cell line MBA-MD468 through adenoviral-mediated gene transfer, and lysates from the adenovirus-infected cells were then applied to glutathione-agarose beads loaded with GST-E2F1 or GST-E7 recombinant proteins purified from bacteria (Fig. 1B). As we have previously reported, GST-E2F1 could pull down RB and RB-N, whereas GST-E7 efficiently pulled down RB but not RB-N (Fig. 1B). In contrast, the RB-K mutant was efficiently pulled down by GST-E7, demonstrating the integrity of the LxCxE-binding pocket in the RB-K protein (Fig. 1B). However, GST-E2F1 did not pull down RB-K, suggesting a critical role of K530 in binding E2F1. As would be expected, the RB-KN was not pulled down by GST-E2F1 or GST-E7 (Fig. 1B). In a reversed GST-pull down experiment, E2F-1 expression was induced in a tetracycline-sensitive SAOS-2 cell line (Fig. 1C) [21]. Cell lysates with overproduced E2F-1 was applied to glutathione beads containing GST-RB or GST-RB-K recombinant proteins expressed in bacteria. The pull-down of E2F1 by GST-RB-K was significantly reduced when compared with that by GST-RB (Fig. 1C).

The defect of RB-K in binding E2F1 was further demonstrated by co-immunoprecipitation (Fig. 1D) and transcription-repression (Fig. 1E). The E2F1-inducible SAOS-2 cells were infected with adenovirus expressing GFP, RB, RB-K, RB-N or RB-KN, followed by the induction of E2F-1 and immunoprecipitation with anti-RB (Fig. 1D). Each of the RB proteins was expressed to similar levels (Fig. 1D, lower panel). The induction of E2F-1 was also similar among the different cultures of infected SAOS-2 cells (Fig 1D, upper panel). The co-immunoprecipitation of E2F-1 was observed with RB and RB-N but not with RB-K or RB-KN (Fig. 1D, middle panel). The C-terminal region of E2F1 is required for its transcriptional activation function, which is inhibited upon the binding of RB [22], [23]. To measure this trans-activation function of E2F1, a Gal4-E2F1 fusion protein was used to drive the expression of a luciferase reporter under the control of tandem Gal4 DNA binding sites [22]. With this well-established co-transfection assay, Gal4-E2F1 stimulated the reporter expression by 300 fold (Fig. 1E). RB reduced the Gal4-E2F1 activity by 3 fold, whereas RB-K did not affect the Gal4-E2F1 activity consistent with the defective binding. Taken together, these results are consistent with the X-ray crystal structure and support the importance of Lysine-530 in the interaction between RB and the C-terminal peptide in E2F1.

### RB-K530A binds to E2F1-DP1

**Figure 2:**
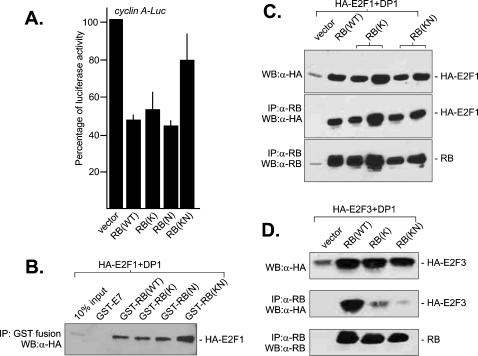
A) Transcription repression. The *Cyclin A-Luc* reporter plasmid contains a firefly luciferase expression cassette preceded with a fragment from the human *Cyclin A* promoter containing E2F-binding site [24]. The reporter was transfected into SAOS-2 cells with the indicated RB expression plasmid and the control Renilla luciferase reporter. The normalized firefly luciferase activity from vector co-transfected cells was set at 100%. B) GST pull-down assay. Lysates from 293T cells expressing HA-tagged E2F1 and DP1 were incubated with the indicated GST-RB and GST-E7 proteins immobilized on glutathione Sepharose. The HA-E2F1 in the bound fraction was resolved by SDS-PAGE and detected by immunoblotting with an anti-HA antibody (α-HA). C) Co-immunoprecipitation. The RB-deficient C33A cells were co-transfected with HA-E2F1, DP1 and the indicated RB expression plasmids. Whole cell lysates were incubated with anti-RB antibody (α-RB), and the co-immunoprecipitated E2F1 examined by immunoblotting with an anti-HA antibody (α-HA). D) GST-pull down assay with E2F3. Lysates from 293T cells expressing HA-tagged E2F3 and DP1 were incubated with the indicated GST-RB and GST-E7 proteins immobilized on glutathione Separose. The amount of E2F3 in the bound fraction was determined by immunoblotting with anti-HA antibody (α-HA).

It is well established that RB can inhibit E2F-dependent transcription through two mechanisms. RB binds the C-terminal trans-activation domain of E2F1 to inhibit transcription [23]. The RB-E2F complex also interacts with other transcription co-repressors such as histone deacetylases or methylases to inhibit transcription [23]. Although RB-K is defective in inhibiting the trans-activation activity of E2F1 (Fig. 1E), it was competent in repressing transcription from an E2F-regulated promoter (Fig. 2A). Previous studies have established the *Cyclin A* promoter to contain E2F-binding site and the promoter activity is repressed by the RB-E2F complex [24]. With a luciferase reporter driven by the *Cyclin A* promoter, it can be shown that RB, RB-K and RB-N are each capable of transcription repression (Fig. 2A). The RB-mediated repression was dependent on E2F, because mutations of the E2F-binding sequences in the *Cyclin A* promoter abolished the RB-mediated transcription repression (not shown). These results suggest that RB-K can still interact with E2F, although not through binding to the E2F C-terminal region.

It has been reported that the RB C-region contains a second binding site for the E2F/DP heterodimer [9]. We therefore determined if RB-K retained the ability to interact with E2F/DP1. A HA-tagged E2F1 was co-expressed with DP1 in 293T cells, and the cell lysates were then incubated with five different GST-fusion proteins- GST-E7, GST-RB, GST-RB-K, GST-RB-N, and GST-RB-KN (Fig. 2B). Under conditions of E2F1 and DP1 co-expression, we found that each of the GST-RB fusion protein were capable of efficient pull down of E2F1. This is in contrast to the result shown in Fig. 1C, when E2F1 was expressed alone without DP1. We also performed co-immunoprecipitation experiments where HA-E2F1, DP1, and RB were transiently co-expressed in the RB-deficient C33A cervical carcinoma cells (Fig. 2C). The interaction between RB, RB-K and RB-KN with E2F1 was consistently observed in independently transfected cells (Fig. 2C), contrasting the result shown in Fig. 1D. It has been reported that the RB C-region preferentially binds E2F1/DP1 and exhibits a much weaker interaction with E2F3/DP1 [9]. When RB, E2F3 and DP1 were transiently co-expressed in 293 cells, we found E2F3 to co-immunoprecipitate with RB but the interaction was much weaker with RB-K (Fig. 2D). This result is consistent with previous reports that RB interacts with E2F3 mostly through its E2F-peptide binding pocket at the A/B interface that is inactivated by the K530A mutation.

### RB-KN lacks growth suppression activity

**Figure 3:**
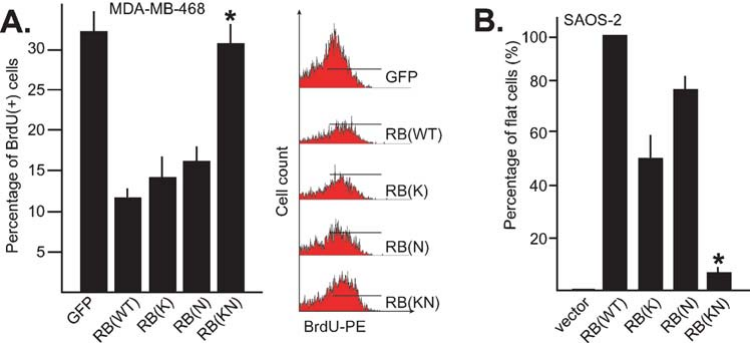
A) BrdU incorporation. MDA-MB468 cells were infected with the indicated recombinant adenoviruses encoding each of the indicated proteins. Infected cells were allowed to incorporate BrdU over a period of 14 hours before they were fixed and stained with phycoerythrin (PE)-conjugated monoclonal antibody against BrdU. The fraction of BrdU-positive cells in each sample was measured by flow cytometry (FACS). The levels of BrdU incorporation in GFP-virus and RB-KN-virus infected cells were statistically similar by student *t*-test (*) from three independent experiments. Representative FACS profiles with the BrdU-positive gates shown are displayed to the right of the histogram. B) Flat cell formation. SAOS-2 cells transfected with plasmids expressing neomycin resistance and each of the indicated RB proteins were cultured in G418 for two weeks. The giant flat cells were stained with crystal violet and their numbers counted under a dissection microscope. The number of flat cells induced by RB in each of four independent experiments was set at 100%. The means and standard errors of relative flat cell induced by vector, RB-K, RB-N and RB-KN from four experiments are shown. Student *t*-test showed the number of flat cells found in RB-KN-transfected cultures was significantly different (* p<0.002) from that in RB-transfected cultures.

We next measured the growth suppression activity of RB mutants by a short-term assay of BrdU-incorporation and a long-term assay of flat cell formation. In the short-term assay, MDA-MB468 cells were infected with adenovirus expressing GFP, RB, RB-K, RB-N or RB-KN and then labeled with BrdU (Fig. 3A). Relative to cells infected with the control GFP-virus, cells infected with RB-, RB-K-, or RB-N-virus showed reduced BrdU incorporation (Fig. 3A). Cells infected with RB-KN, on the other hand, incorporated BrdU to a level that was indistinguishable from the GFP-virus infected cells (Fig. 3A).

In the long-term assay, we used the well-established induction of senescence by RB in the RB-deficient human SAOS-2 cells. Ectopic expression of RB induces the formation of large flat cells that are metabolically active but do not undergo cell division [20]. In RB-transfected cultures, the flat cell numbers ranged from 200–400 per plate in four independent experiments. In mock- transfected cultures, 0–4 flat cells were found per plate. In RB-N-transfected cultures, 120–220 flat cells were found, and in RB-K-transfected cells, 80–170 flat cells were counted. The relative flat cell counts from the four experiments are shown in Fig. 3B. Only 10–20 flat cells were found in cultures transfected with the RB-KN mutant, showing a statistically significant reduction in the flat-cell induction activity of this mutant (Fig. 3B). Because the levels of protein expression through adenovirus infection were higher than those from DNA transfection, we also compared the effect of RB mutants on BrdU incorporation in transfected SAOS-2 cells. The result was consistent with the flat cell assay: inhibition of BrdU incorporation by RB-K was less efficient than by RB, whereas RB-KN did not inhibit BrdU incorporation (not shown). These results show that the RB-K or the RB-N mutant retains growth suppression activity, but the RB-KN mutant is defective in growth suppression, consistent with its defect in repressing transcription from the *Cyclin A* promoter (Fig. 2A).

### RB-KN inhibits apoptosis

Because RB-KN is defective in growth suppression, this mutant provides an opportunity to test the hypothesis that the anti-apoptotic function of RB is secondary to growth arrest.

**Figure 4:**
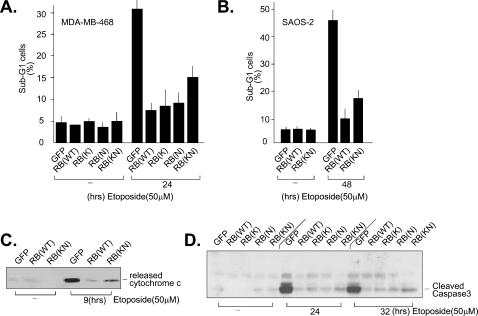
A) Etoposide-induced DNA fragmentation in MDA-MB468 cells. Cells were infected with recombinant adenovirus expressing the indicated proteins. Infected cells were treated with 50 µM etoposide or vehicle control for 24 hr, fixed, stained with propidium iodide and DNA fragmentation (sub-G1 DNA content) measured by FACS. B) Etoposide-induced DNA fragmentation in SAOS-2 cells. The same experiment as in (A) except the duration of drug treatment was for 48 hours. C) Etoposide-induced cytochrome *c* release. MDA-MB468 cells infected with the indicated recombinant adenovirus treated with 50 µM etoposide or vehicle control. At 9 hours after drug addition, cells were homogenized and the mitochondria were separated from the cytosolic fraction by centrifugation. The levels of cytochrome *c* in the post-mitochondrial supernatants were determined by immunoblotting. D) Etoposide-induced caspase 3 cleavage. MDA-MB468 cells infected with the indicated recombinant adenovirus were treated with 50 µM etoposide. Cells were harvested at the indicated time points and the levels of cleaved caspase-3 determined by immunoblotting with antibodies specific to the cleaved caspase-3.

The RB-deficient MDA-MB468 breast cancer cells were infected with adenovirus expressing GFP, RB, RB-K, RB-N, or RB-KN. The infected cells were then treated with etoposide. Apoptosis was assessed by DNA fragmentation (sub-G1 DNA content) (Fig. 4A), cytochrome *c* release (Fig. 4C), and the cleavage of pro-caspase 3 (Fig. 4D). Etoposide treatment led to a significant increase in DNA fragmentation (assessed by the sub-G1 DNA content) in GFP-virus infected cells (Fig. 4A). The increase in sub-G1 DNA content was negligible in etoposide-treated cells that were infected with RB, RB-K, or RB-N virus (Fig. 4A). This result supports the conclusion that RB-mediated growth arrest confers resistant to genotoxin-induced apoptosis. Interestingly, however, the RB-KN-virus infected cells also displayed reduced DNA fragmentation (Fig. 4A), despite the continued cell cycle progression (Fig. 3A).

Exposure to etoposide induced cytochrome *c* release in GFP-virus infected cells (Fig. 4C). Activation of this critical step in the intrinsic apoptotic pathway by etoposide was reduced in cells expressing RB or RB-KN (Fig. 4C). The etoposide-induced cleavage of pro-caspase-3 was reduced in MDA-MB438 cells expressing RB, RB-K, or RB-N (Fig. 4D). This proteolytic processing of caspase-3 was also reduced in cells expressing RB-KN (Fig. 4D). The anti-apoptotic effect of RB-KN was observed in SAOS-2 cells as well (Fig. 4B), showing that RB-KN could inhibit apoptosis without suppressing cell growth in two different RB-deficient cell lines.

### RB retains anti-apoptotic function in proliferating cells

**Figure 5:**
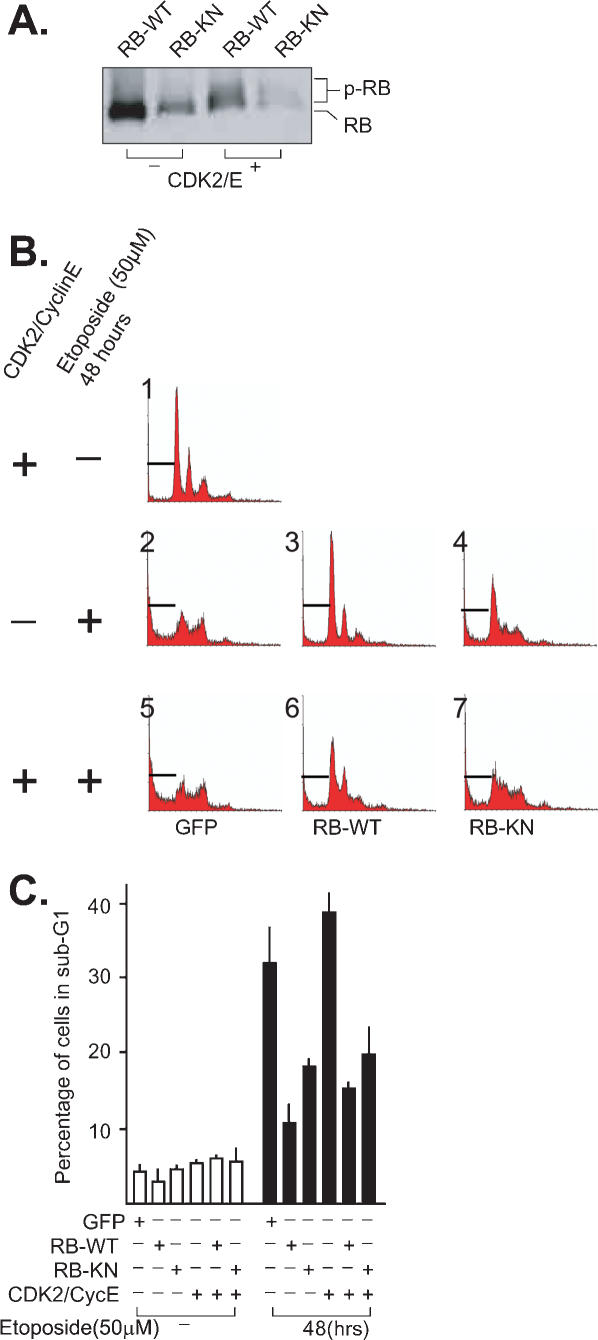
A) RB phosphorylation by Cdk2/Cyclin E in SAOS-2 cells. Cells were infected with recombinant adenovirus expressing RB or RB-KN, in the absence or presence of co-infection with recombinant adenovirus expressing Cdk2 and Cyclin E. The electrophoresis mobility of RB was examined by immunoblotting of whole cell lysates with anti-RB. B) Representative FACS profiles. SAOS-2 cells infected with the indicated recombinant adenoviruses were treated with 50 µM etoposide, harvested 48 hours later, fixed, stained with propidium iodide and the DNA content analyzed by FACS. The gate for sub-G1 DNA fraction is shown in each profile. C) Summary of DNA fragmentation results. The sub-G1 DNA content was determined by FACS analysis as illustrated in panel B. The values are mean and standard errors from three independent experiments.

To determine whether wild type RB has anti-apoptotic function in proliferating cells, we took note of the results that RB-mediated growth arrest can be overridden by the co-expression of cyclins [25], [26]. We therefore co-infected SAOS-2 cells with adenovirus expressing RB and Cdk2/Cyclin E. As would be expected, co-expression of RB with Cdk2/Cyclin E led to RB phosphorylation, indicated by the altered electrophoresis mobility in SDS-PAGE (Fig. 5A).

SAOS-2 cells were infected with adenovirus expressing GFP, RB or RB-KN alone, or in combination with adenovirus expressing Cdk2/Cyclin E. Infected cells were then exposed to etoposide, harvested 48 hours later for FACS analyses of DNA content (Fig. 5B & C). The expression of Cdk2/Cyclin E with GFP caused an increase in the S-phase DNA content, but not an increase in sub-G1 DNA (Fig. 5B, #1). Etoposide-induced increase in sub-G1 DNA content was similar in GFP-expressing cells that expressed Cdk2/Cyclin E (Fig. 5B, #5) or not (Fig. 5B, #2) (see also Fig. 5C). Expression of RB or RB-KN protected SAOS-2 cells from etoposide-induced DNA fragmentation (Fig. 5B, #3 and #4) (Fig. 5C). Co-expression with Cdk2/Cyclin E increased the S-phase fraction of etoposide-treated, RB-positive, SAOS-2 cells (Fig. 5B, #6); yet, it did not override the anti-apoptotic function of RB (Fig. 5C). Co-expression with Cdk2/Cyclin E did not affect either the S-phase or the sub-G1 fraction of etoposide treated, RB-KN-expressing, SAOS-2 cells (Fig. 5B, #7 and Fig. 5C). These results suggest wild-type RB can also reduce apoptosis under condition in which its growth suppression function is impaired.

## Discussion

The anti-proliferation function of RB is mediated by the repression of E2F-dependent transcription of cell cycle genes [4], [27]. RB can inhibit E2F-dependent transcription by two mechanisms: either through the direct binding to the C-terminal trans-activation domain of E2F or through the assembly of transcription repressor complexes at E2F-regulated promoters [4], [23], [27]. The RB-K mutant does not bind to the C-terminal trans-activation domain of E2F and it does not inhibit the trans-activation function of E2F1. However, RB-K still interacts with E2F1/DP1, most likely via the C-terminal E2F/DP-binding site [9], of which the X-ray crystal structure has recently been reported [28]. The RB-N mutant retains both E2F-binding pockets, i.e., at the A/B interface and at the C-terminal region [8], accounting for its ability to inhibit cell proliferation. Because the RB-KN double mutant is defective in transcription repression, we can conclude that an intact LxCxE-binding pocket is required for RB-K to repress transcription. Our findings suggest RB-K may inhibit proliferation through the assembly of transcription repression complexes, whereas RB-N is likely to directly inhibit the trans-activation function of E2F. It thus appears that either of the two established transcriptional repression mechanisms is sufficient for RB to inhibit cell proliferation.

Previous studies have shown that RB-deficient cells are hypersensitive to DNA damage-induced apoptosis, and that expression of wild-type RB in RB-deficient cells can rescue this hypersensitivity [11], [13], [27]. Furthermore, induced expression of a phosphorylation-site-mutated PSM-RB causes G1 arrest to protect cells from DNA damage-induced apoptosis [15]. The coordinated suppression of proliferation and apoptosis by RB can be explained by its inhibition of E2F-dependent transcription [27]. This study of the RB-K, RB-N and RB-KN mutants, however, has shown that RB can also inhibit apoptosis in proliferating cells. At present, we do not understand the mechanism through which RB-KN inhibits etoposide-induced apoptosis. RB-KN retains the C-terminal E2F1/DP binding site, and may conceivably inhibit a subset of E2F1/DP-regulated promoters (other than that of *Cyclin A*) to inhibit apoptosis. Alternatively, RB-KN may sequester pro-apoptotic factors other than E2F1, for example, the nuclear ABL tyrosine kinase that has been shown to promote DNA damage-induced apoptosis [29], [30]. Because RB-E2F1 and RB-ABL interactions are disrupted by RB-phosphorylation in proliferating cells [31], [32], the anti-apoptotic activity associated with phosphorylated RB or RB-KN may not be accounted for by the inhibition of E2F-1 and ABL. A recent study has shown that RB can stimulate DNA repair [33], [34], which will enhance cell survival under conditions of genotoxic stress. The RB-KN mutant should facilitate future studies to elucidate the growth suppression-independent anti-apoptotic function of RB.
